# 

**Published:** 1905-07-15

**Authors:** 


### CONTENTS.

Event and Comment - - - - - - - - - - - 325

Amalgams and Their Peculiarities, - - By M. L. Ward, D.D.S. 331

Our Annual Income as an Element in Dental Pro3ress, By B. J. Cigrand, M.S., D.D.S. 347

Announcements - - - - - - - - - - - - 361

Biblio3raphy - - - - - - - - - - - - - 363

Obituary - - - - - - - - - - - - - 364

Indents - - - - - - - - - - - - - 365

